# Clinical and genomic diversity of *Treponema pallidum* subspecies *pallidum* to inform vaccine research: an international, molecular epidemiology study

**DOI:** 10.1016/S2666-5247(24)00087-9

**Published:** 2024-09

**Authors:** Arlene C Seña, Mitch M Matoga, Ligang Yang, Eduardo Lopez-Medina, Farhang Aghakhanian, Jane S Chen, Everton B Bettin, Melissa J Caimano, Wentao Chen, Jonny A Garcia-Luna, Christopher M Hennelly, Edward Jere, Yinbo Jiang, Jonathan J Juliano, Petra Pospíšilová, Lady Ramirez, David Šmajs, Joseph D Tucker, Fabio Vargas Cely, Heping Zheng, Irving F Hoffman, Bin Yang, M Anthony Moody, Kelly L Hawley, Juan C Salazar, Justin D Radolf, Jonathan B Parr

**Affiliations:** a Department of Medicine, Division of Infectious Diseases, Institute for Global Health and Infectious Diseases, University of North Carolina at Chapel Hill, Chapel Hill, NC, USA; b UNC Project Malawi, Tidziwe Centre, Lilongwe, Malawi; c Dermatology Hospital, Southern Medical University, Guangdong Provincial Center for Skin Diseases and STD Control, Guangzhou, China; d Centro Internacional de Entrenamiento e Investigaciones Medicas, Campus de la Universidad Icesi, Cali, Colombia; e Department of Pediatrics, Universidad del Valle, Cali, Colombia; f Institute for Global Health and Infectious Diseases, Infectious Diseases Epidemiology and Ecology Laboratory, University of North Carolina at Chapel Hill, Chapel Hill, NC, USA; g Department of Health Behavior, Gillings School of Global Public Health, University of North Carolina at Chapel Hill, Chapel Hill, North Carolina, USA; h Department of Medicine, UConn Health, Farmington, CT, USA; i Department of Pediatrics, UConn Health, Farmington, CT, USA; j Department of Immunology, UConn Health, Farmington, CT, USA; k Connecticut Children's, Hartford, CT, USA; l BSL-3 Laboratory, Guangdong Provincial Key Laboratory of Tropical Disease Research School of Public Health, Southern Medical University, Guangzhou, China; m Universidad Icesi, Cali, Colombia; n Division of Dermatology, Department of Internal Medicine, School of Medicine, Universidad del Valle, Cali, Colombia; o Department of Biology, Faculty of Medicine, Masaryk University Brno, Czech Republic; p Department of Pediatrics, Division of Infectious Diseases, Duke University Medical Center, Durham, NC, USA; q Department of Integrative Immunology, Duke University Medical Center, Durham, NC, USA; r Duke Human Vaccine Institute, Durham, NC, USA

## Abstract

**Background:**

The increase in syphilis rates worldwide necessitates development of a vaccine with global efficacy. We aimed to explore *Treponema pallidum* subspecies *pallidum* (TPA) molecular epidemiology essential for vaccine research by analysing clinical data and specimens from early syphilis patients using whole-genome sequencing (WGS) and publicly available WGS data.

**Methods:**

In this multicentre, cross-sectional, molecular epidemiology study, we enrolled patients with primary, secondary, or early latent syphilis from clinics in China, Colombia, Malawi, and the USA between Nov 28, 2019, and May 27, 2022. Participants aged 18 years or older with laboratory confirmation of syphilis by direct detection methods or serological testing, or both, were included. Patients were excluded from enrolment if they were unwilling or unable to give informed consent, did not understand the study purpose or nature of their participation, or received antibiotics active against syphilis in the past 30 days. TPA detection and WGS were conducted on lesion swabs, skin biopsies, skin scrapings, whole blood, or rabbit-passaged isolates. We compared our WGS data to publicly available genomes and analysed TPA populations to identify mutations associated with lineage and geography.

**Findings:**

We screened 2802 patients and enrolled 233 participants, of whom 77 (33%) had primary syphilis, 154 (66%) had secondary syphilis, and two (1%) had early latent syphilis. The median age of participants was 28 years (IQR 22–35); 154 (66%) participants were cisgender men, 77 (33%) were cisgender women, and two (1%) were transgender women. Of the cisgender men, 66 (43%) identified as gay, bisexual, or other sexuality. Among all participants, 56 (24%) had HIV co-infection. WGS data from 113 participants showed a predominance of SS14-lineage strains with geographical clustering. Phylogenomic analyses confirmed that Nichols-lineage strains were more genetically diverse than SS14-lineage strains and clustered into more distinct subclades. Differences in single nucleotide variants (SNVs) were evident by TPA lineage and geography. Mapping of highly differentiated SNVs to three-dimensional protein models showed population-specific substitutions, some in outer membrane proteins (OMPs) of interest.

**Interpretation:**

Our study substantiates the global diversity of TPA strains. Additional analyses to explore TPA OMP variability within strains is vital for vaccine development and understanding syphilis pathogenesis on a population level.

**Funding:**

US National Institutes of Health National Institute for Allergy and Infectious Disease, the Bill & Melinda Gates Foundation, Connecticut Children’s, and the Czech Republic National Institute of Virology and Bacteriology.

## Introduction

*Treponema pallidum* subspecies *pallidum* (TPA), the causative agent of syphilis, is highly invasive and can cause protean clinical manifestations, adverse pregnancy outcomes, and increased HIV transmission.^1^ In 2020, WHO estimated that there were 22ꞏ3 million prevalent syphilis cases and 7ꞏ1 million incident cases worldwide.^2^ Development of a syphilis vaccine will be key to stemming this epidemic.^3^

**Research in context**

**Evidence before this study**

Several studies have conducted whole genome-sequencing of *Treponema pallidum* subspecies *pallidum* (TPA) and contributed to our understanding of the global distribution of circulating strains. However, analyses of participant specimens for the purpose of evaluating TPA clinical and genetic diversity to inform syphilis vaccine development have been scarce. We conducted a PubMed search from database inception up to May 25, 2023, using the search terms “*Treponema pallidum*” and “genome sequencing” and “global”, and identified 15 articles with relevant TPA whole-genome sequencing data from samples collected worldwide. Most TPA genome sequences have been reported from Europe, North America, and Australia. One study assessed TPA genome sequencing and the variability of TPA vaccine candidate genes from six continents but had little clinical data which included only the stage of syphilis.

**Added value of this study**

In this multicentre, cross-sectional study of well characterised participants with early syphilis, we evaluated clinical characteristics associated with TPA strains, compared them to global strains published to date, and analysed single nucleotide variants among infecting strains. Our study adds more than 160 genetically and geographically diverse SS14-lineage and Nichols-lineage TPA genomes to the literature from under-represented areas including Africa, Asia, and South America. There was a predominance of the SS14-lineage globally, with distinct geographical associations shown for several Nichols-lineage populations. We did not identify differences in the distribution of TPA strains based on demographic, behavioural, or clinical characteristics of participants. We identified common, lineage-informative mutations and mapped them to three-dimensional models of periplasmic and outer membrane proteins (OMPs) that might pertain to disease pathogenesis, protective immunity, and syphilis vaccine design.

**Implications of all the available evidence**

TPA genomic analyses from participants with early syphilis enrolled through our global consortium support previously reported genetic population structure, with eight distinct populations worldwide and SS14-lineage predominance. We identified lineage-informative mutations that varied by TPA population, some of which localised to OMP extracellular loops and segregated by geography. Our findings confirm the importance of TPA sampling from different clinical sites to facilitate the development of a syphilis vaccine with global efficacy.

Long-standing problems with in-vitro TPA cultivation have complicated syphilis research.^4^ Fortunately, advances in whole-genome sequencing (WGS) performed directly from biological specimens have increased the number of available genomes and our understanding of TPA’s genetic diversity.^5^, ^6^, ^7^, ^8^ Studies have shown two deep-branching TPA phylogenetic lineages that are differentiated by their relationship to the Nichols or SS14 reference strains.^5^, ^6^, ^7^, ^8^ Mapping of single-nucleotide variants (SNVs) to structural models for TPA outer membrane proteins (OMPs) can identify regions of sequence variability relevant to vaccine design and pathogenesis.^9^

Most TPA genomic sequences originated from strains circulating in high-income countries,^5^, ^6^, ^7^, ^8^ or from retrospective analyses with little clinical data. We hypothesised that TPA genetic diversity might exist between populations with differing demographic and clinical characteristics, and geography from low-income and middle-income countries (LMICs). Thus, we developed a global consortium to collect individual-level data and biological specimens for WGS analyses to explore strain diversity. We aimed to evaluate the characteristics associated with TPA strains, compare them to global strains published to date, and analyse SNVs among strains to inform vaccine development.

## Methods

### Study design and participants

We conducted a multicentre, observational, cross-sectional study by recruiting and enrolling a convenience sample of patients with early syphilis (primary, secondary, and early latent syphilis) who presented to the Dermatology Hospital of Southern Medical University in Guangzhou, China; a network of approximately 100 health-care institutions that refer to the Centro Internacional de Entrenamiento e Investigaciones Medicas (CIDEIM) in Cali, Colombia; the sexually transmitted infections clinic at Bwaila Hospital in Lilongwe, Malawi; and the UNC Infectious Disease Clinic in Chapel Hill, NC, USA, between Nov 28, 2019, and May 27, 2022.

Screening criteria for participants included age 18 years or older and one of the following: suspected primary syphilis with ulcerative lesions (eg, chancres), secondary syphilis with mucocutaneous lesions (eg, rashes or condyloma lata), or early latent syphilis with seroconversion or early syphilis exposure within the past 12 months (appendix 1 pp 5–6). Additional enrolment criteria included confirmation of primary syphilis with darkfield microscopy (DFM), PCR, or syphilis serology; or the confirmation of secondary syphilis or early latent syphilis with positive non-treponemal and treponemal antibody tests. Patients were excluded from enrolment if they were unwilling or unable to give informed consent, did not understand the study purpose or nature of their participation, or received antibiotics active against syphilis in the past 30 days. Participants provided written informed consent.

The study was approved by the institutional review boards (IRBs) at the Dermatology Hospital of Southern Medical University in Guangzhou (IRB protocol Guangdong Dermatology Hospital Lunli Shencha-20181202 [R3]), CIDEIM in Cali (IRB 163, protocol 1289), the National Health Sciences Research Committee Ministry of Health in Lilongwe (IRB approval 2252), and the University of North Carolina at Chapel Hill (IRB protocol 19-0311).

### Procedures

Sexual histories, physical examinations, and HIV or sexually transmitted infection testing were conducted as per routine care. Participants underwent screening using DFM from lesions, non-treponemal, or rapid treponemal antibody tests. Urine pregnancy testing was performed for women of childbearing age.

Participants underwent data collection, photography of lesions, and specimen collection via lesion swabs, punch skin biopsies, or skin scrapings of rashes. Multiple swabs were collected if participants had more than one lesion. Lesion swabs, skin biopsies, or skin scrapings were collected in DNA/RNA Shield reagent (Zymo Research, Irvine, CA, USA). Approximately 20 mL of whole blood was collected for serum and plasma; the China and Colombia sites collected additional blood for peripheral blood mononuclear cells. Lesion exudates and whole blood were collected for rabbit infectivity testing^10^ in Guangzhou (appendix 1 p 22).

After specimen collection, all participants received treatment according to national guidelines. Behavioural counselling and partner services were provided per routine care.

Demographic and clinical data, sexual histories, laboratory results, and treatment were documented in case report forms (CRFs). Participant gender was self-reported as either cisgender male, cisgender female, transgender male, transgender female, or other. English CRFs (appendix 1 pp 11–21) were translated into local languages. CRF data and photographs were uploaded into a secure Research Electronic Data Capture database.

We performed quantitative PCR (qPCR) targeting the TPA polymerase I gene (*polA*;*tp0105*) on DNA extracted from lesion swabs, skin biopsies, skin scrapings, and whole blood.^11^ Any qPCR-positive sample with a copy number greater than zero was considered TPA positive. qPCR was also conducted on rabbit-passaged isolates from primary syphilis lesion exudates and secondary syphilis whole blood samples from Guangzhou (appendix 1 p 22).

TPA WGS was performed on specimens from enrolled participants and others that were qPCR positive including (1) DFM-negative lesion swabs from participants screened in Lilongwe, and (2) primary syphilis and secondary syphilis lesions from participants enrolled in Cali in separate IRB-approved studies (IRB reference numbers 1281, 1289, 1315; appendix 1 pp 21–22). In general, available specimens with 40 or more *polA* copies per μL underwent TPA enrichment and WGS (appendix 1 pp 22–23). TPA enrichment was performed using parallel, pooled whole-genome amplification^12^ and custom 120-nucleotide RNA oligonucleotide baits (SureSelect XT Low Input in Chapel Hill or SureSelect XTHS2 in Guangzhou; Agilent Technologies, Santa Clara, CA, USA). Pooled, TPA-enriched libraries were sequenced using MiSeq or NovaSeq platforms (Illumina, San Diego, CA, USA) with paired-end, 150 bp reads.

Sequencing data, along with publicly available data from geographically diverse locations,^8^ were analysed using an adaptation of our bioinformatic pipeline (appendix 1 pp 22–24).^13^ After read trimming, alignment, filtering, and variant calling, the consensus genomes were aligned. Repetitive and putative recombination regions were masked before construction of maximum likelihood trees. Clades and subclades were manually assigned to facilitate comparison with published analyses;^8^ clustering was evaluated using Bayesian modelling with partitioning informed by maximum likelihood phylogeny. We identified macrolide-resistant strains using stringent competitive mapping.^6^^,^^14^ To evaluate population structure, we analysed our samples alongside publicly available TPA genomes downloaded from Sequence Read Archive (appendix 2 p 1), using principal component analysis and population structure estimation using Bayesian modelling to identify TPA populations. SNVs were mapped to protein structural models of genes with high frequency, population-informative mutations.

### Data analyses

Participant characteristics are described with percentages, medians, and IQRs. Missing data were annotated in subsequent descriptive analyses. The analyses of TPA qPCR were based on results from the local laboratories, with the exception of Malawi, which had all TPA qPCR testing performed at UNC. For participants with multiple TPA qPCR results per sample type, overall TPA qPCR sample results were classified as follows: if at least one individual sample result was more than 0 copies per μL, the overall result was classified as positive with a quantitative result as the geometric mean of all values with more than 0 copies per μL. Otherwise, the overall sample result was classified as negative (0 copies per μL). All analyses were performed using R (v4.1.2).

### Role of the funding source

The funders of the study had no role in study design, data collection, data analysis, data interpretation, or writing of the report.

## Results

**Figure 1:**
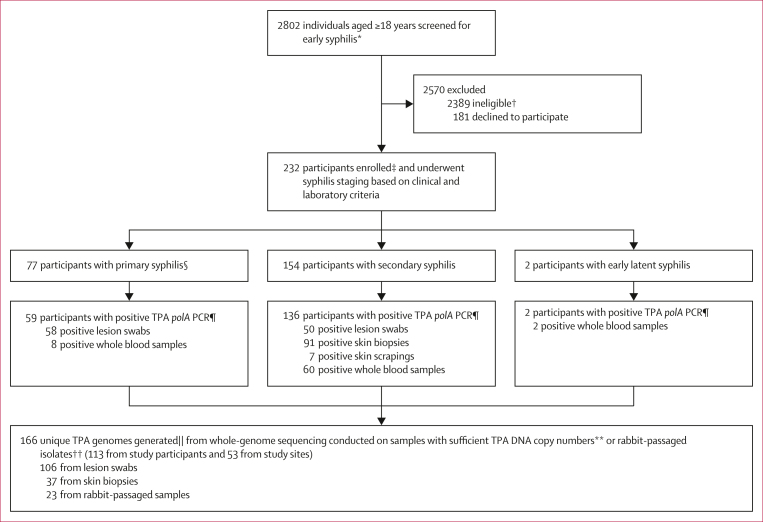
Screening, enrolment, and specimen testing algorithm for participants with early syphilis in this study TPA=*Treponema pallidum s*ubspecies *pallidum*. ∗Screening based on suspected primary, secondary, or early latent syphilis among patients from clinical sites in China, Colombia, Malawi, and USA. †Includes individuals who refused screening procedures in Malawi. ‡One participant was enrolled twice, initially with secondary syphilis and then with primary syphilis. §One participant initially staged with secondary syphilis had a PCR-positive skin biopsy but, upon later review, was restaged as having a healed primary lesion. ¶Each participant can contribute multiple specimen types. ||166 total of unique genomes generated, which includes sequences from fully enrolled study participants; 43 participants from Malawi consented for screening who had darkfield-negative lesions but were PCR-positive, and a convenience sample of 10 individuals from Colombia enrolled in a longitudinal study with available specimens. ∗∗In general, available specimens with 40 or more *polA* copies per μL underwent TPA enrichment and whole-genome sequencing. ††Lesion exudates from primary syphilis and whole blood from secondary syphilis were passaged through rabbits to enrich for treponemes.

Between Nov 28, 2019, and May 27, 2022, we screened 2802 individuals with suspected syphilis (figure 1). We enrolled 232 unique participants, including one participant enrolled in 2020 with secondary syphilis and enrolled in 2021 with primary syphilis. Of the 233 total participants, 77 (33%) had primary syphilis, 154 (66%) had secondary syphilis, and two (1%) had early latent syphilis (table). Only two participants were enrolled from the US site, which halted enrolment from Jan 27 to March 19, 2020 due to the COVID-19 pandemic.

**Table:** Characteristics of participants by site of enrolment and *Treponema pallidum* subspecies *pallidum* WGS results in the multicentre study

	China (n=69)	Colombia (n=74)	Malawi (n=88)	USA (n=2)	Total (n=233)	WGS (n=113)
**Demographic characteristics**						
Age, years	27 (22–32)	28 (22–39)	28 (22–33)	(51–55)∗	28 (22–35)	26 (22–31)
Gender						
Cisgender women	11 (16%)	20 (27%)	46 (52%)	0	77 (33%)	37 (33%)
Cisgender men	58 (84%)	53 (72%)	42 (48%)	1 (50%)	154 (66%)	75 (66%)
Transgender women	0	1 (1%)	0	1 (50%)	2 (1%)	1 (1%)
Sexual orientation						
Heterosexual	45 (65%)	31 (42%)	86 (98%)	0	162 (70%)	86 (76%)
Gay, bisexual, or other	23 (33%)	43 (58%)	0	1 (50%)	67 (29%)	25 (22%)
Unknown or unwilling to state	1 (1%)	0	2 (2%)	1 (50%)	4 (2%)	2 (2%)
Race or ethnicity						
African American	0	3 (4%)	0	1 (50%)	4 (2%)	0
African American; Hispanic or Latino	0	14 (19%)	0	0	14 (6%)	5 (4%)
Asian	69 (100%)	0	0	0	69 (30%)	52 (46%)
Black African	0	0	88 (100%)	1 (50%)	89 (38%)	47 (42%)
Hispanic or Latino	0	48 (65%)	0	0	48 (21%)	8 (7%)
White; Hispanic or Latino	0	9 (12%)	0	0	9 (4%)	1 (1%)
**Behavioural characteristics**						
Sexual partners in past year	3 (2–5)	2 (1–4)	1 (1–2)	(3–4)∗	2 (1–3)	2 (1–3)
New sexual partners in past year	1 (1–3)	1 (0–3)	1 (0–1)	(3–4)∗	1 (0–2)	1 (0–3)
Exchanged goods, money, or favours for sex with a partner in the past year†	13 (19%)	11 (15%)	31 (35%)	1 (50%)	56 (24%)	34 (30%)
Previous intravenous drug use	0	1 (1%)	0	0	1 (<1%)	0
**Medical history**						
HIV co-infection						
Not living with HIV	68 (99%)	37 (50%)	72 (82%)	0	177 (76%)	98 (87%)
Living with HIV	1 (1%)	37 (50%)	16 (18%)	2 (100%)	56 (24%)	15 (13%)
New diagnosis, no ART‡	0	2 (5%)	5 (31%)	0	7 (13%)	3 (20%)
Known diagnosis, no ART‡	0	5 (14%)	0	0	5 (9%)	0
Known diagnosis, on ART‡	1 (100%)	30 (81%)	11 (69%)	2 (100%)	44 (79%)	12 (80%)
Pregnant	0	5 (7%)	2 (2%)	0	7 (3%)	5 (4%)
History of syphilis or genital ulcer disease	6 (9%)	24 (32%)	7 (8%)	1 (50%)	38 (16%)	7 (6%)
History of other STIs	15 (22%)	17 (23%)	13 (15%)	2 (100%)	47 (20%)	27 (24%)
**Clinical characteristics**						
Syphilis stage						
Primary syphilis§	18 (26%)	15 (20%)	44 (50%)	0	77 (33%)	46 (41%)
Secondary syphilis	51 (74%)	58 (78%)	44 (50%)	1 (50%)	154 (66%)	67 (59%)
Early latent syphilis	0	1 (1%)	0	1 (50%)	2 (1%)	0
Darkfield microscopy¶						
Positive	12 (17%)	5 (7%)	59 (67%)	0	76 (33%)	50 (44%)
Negative	6 (9%)	9 (12%)	18 (20%)	0	33 (14%)	10 (9%)
Equivocal	0	4 (5%)	0	0	4 (2%)	1 (1%)
Not performed	51 (74%)	56 (76%)	11 (12%)	2 (100%)	120 (52%)	52 (46%)
Non-treponemal antibody test						
Negative	9 (13%)	1 (1%)	10 (11%)	0	20 (9%)	6 (5%)
Reactive	60 (87%)	73 (99%)	78 (89%)	2 (100%)	213 (91%)	107 (95%)
Non-treponemal titre||	1:32 (1:16–1:64)	1:32 (1:16–1:64)	1:32 (1:32–1:128)	(1:64–1:128)∗	1:32 (1:16–1:64)	1:32 (1:16–1:64)
Treponemal antibody test∗∗						
Negative	2 (3%)	0	0	0	2 (1%)	0
Positive	67 (97%)	74 (100%)	78 (89%)	2 (100%)	221 (95%)	113 (100%)
Not performed	0	0	10 (11%)	0	10 (4%)	0
Treatment for syphilis						
Benzathine penicillin 2·4 million units intramuscularly in a single dose	10 (14%)	23 (31%)	27 (31%)	2 (100%)	62 (27%)	30 (27%)
Benzathine penicillin 2·4 million units intramuscularly once weekly for 2 weeks	45 (65%)	0	0	0	45 (19%)	37 (33%)
Benzathine penicillin 2·4 million units intramuscularly once weekly for 3 weeks	1 (1%)	50 (68%)	60 (68%)	0	111 (48%)	39 (35%)
Doxycycline 100 mg orally twice daily for 14 days	12 (17%)	0	1 (1%)	0	13 (6%)	7 (6%)
Doxycycline 100 mg orally twice daily for 28 days	1 (1%)	1 (1%)	0	0	2 (1%)	0

Data are n (%) or median (IQR). The data include two enrolments of a participant who enrolled twice in China. ART=antiretroviral therapy. STI=sexually transmitted infections. WGS=whole-genome sequencing.

∗ n=2, both values listed.

† Sex exchanged for goods, money, or favours was assessed for up to five sexual partners from the past year. If no partners named this category was classified as no.

‡ The proportion of participants on ART were estimated using the number of persons living with HIV as the denominator.

§ One participant initially staged with secondary syphilis had a PCR-positive skin biopsy but, upon later review, was restaged as having a healed primary lesion.

¶ Darkfield only performed on participants with genital ulcers, condyloma lata, and mucous patches.

|| One non-treponemal titre was missing among participants enrolled at the China site.

∗∗ At the Malawi site, treponemal antibody testing was only performed for participants with a positive non-treponemal test.

Of the 233 participants, the median age was 28 years (IQR 22–35); 154 (66%) participants were cisgender men, 77 (33%) were cisgender women, and two (1%) were transgender women (table). 66 (43%) of 154 cisgender men reported identifying as gay, bisexual, or other sexuality. Seven (9%) of 77 cisgender women were pregnant at enrolment. 69 (30%) of 233 participants were Asian, 89 (38%) were Black African, and 71 (30%) were Hispanic or Latino. The median number of sexual partners in the past year was two (IQR 1–3), but 56 (24%) of the 233 participants reported exchanging goods, money, or favours for sex in the past year (table). 56 (24%) of 233 participants were newly diagnosed or living with HIV and 44 (79%) of 56 were on antiretroviral therapy. Overall, 38 (16%) of 233 participants reported a history of previous syphilis or genital ulcer disease, and 47 (20%) reported a history of other sexually transmitted infections.

**Figure 2:**
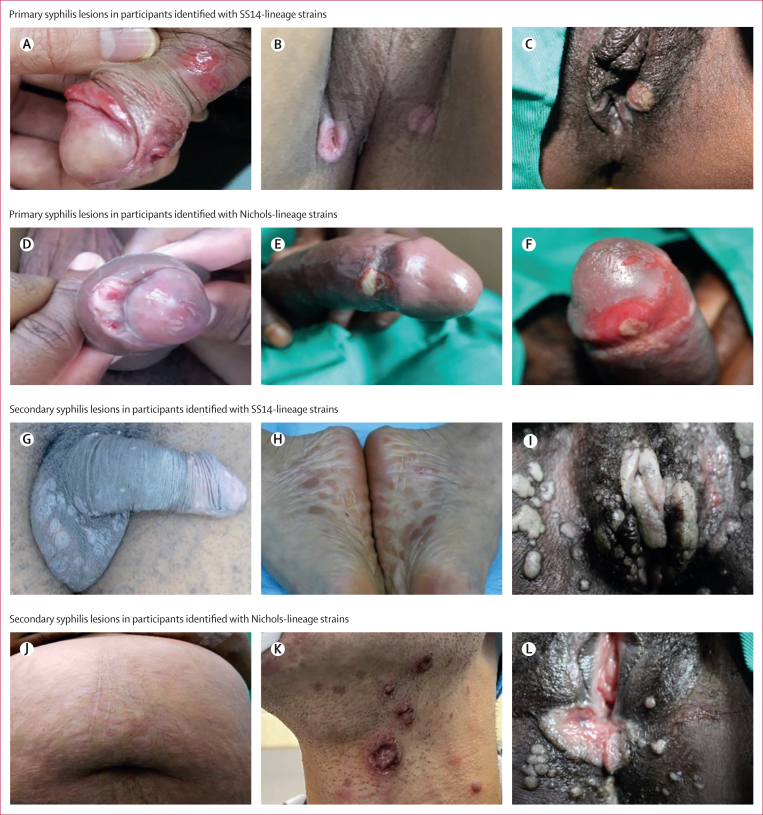
Clinical manifestations among study participants with primary and secondary syphilis, by TPA clade, geographical location, and quantitative PCR result (copies per μL) (A) Multiple, shallow penile ulcers (China; lesion swab = 22 copies per μL). (B) Multiple, deep perineal ulcers (Colombia; lesion swab = 2117 copies per μL). (C) Multiple vulvar chancres (Malawi; 8764 copies per μL). (D) Penile ulcer underneath the foreskin with a purulent discharge (Colombia; lesion swab = 3034 copies per μL). (E) Penile chancres with purulent base (Malawi; lesion swabs = 6897 copies per μL). (F) Penile chancres with purulent base (Malawi; lesion swabs = 2281 copies per μL). (G) Diffuse scaly, macular rash involving testicles (Colombia, skin biopsy = 53 copies per μL). (H) Hyperpigmented macules involving soles (China; skin biopsy = 305 copies per μL). (I) Extensive condyloma lata involving labia and perineum (Malawi; lesion swab = 2281 copies per μL). (J) Erythematous macular rash in pregnancy (Colombia; skin biopsy = 2460 copies per μL). (K) Diffuse rash with crusting lesions consistent with lues maligna (China; skin biopsy = 250 copies per μL). (L) Moist, condyloma lata involving vulvar area and perineum (Malawi; lesion swab = 6897 copies per μL).

DFM was performed in 113 enrolled participants who had lesion swabs, 76 (67%) of which were positive. Among 233 enrolments, 213 (91%) participants had reactive non-treponemal and 221 (95%) participants had reactive treponemal tests. The median non-treponemal titre was 1:32 (IQR 1:8–1:64) for primary syphilis and 1:32 (IQR 1:32–1:64) for secondary syphilis. We observed various clinical manifestations (figure 2), including single and multiple anogenital ulcers in primary syphilis. Secondary syphilis manifestations ranged from maculopapular rashes to ulcerating lesions of lues maligna, and extensive anogenital condyloma lata. The majority (197 [85%] of 233) of participants were TPA qPCR positive from multiple specimen types (figure 1). TPA burdens varied by syphilis stage, clinical manifestations, and clinical sites (figure 2; appendix 2 p 2). Skin biopsies or scrapings and whole blood specimens had lower TPA qPCR geometric mean copy numbers than lesion swabs (appendix 2 p 2).

**Figure 3:**
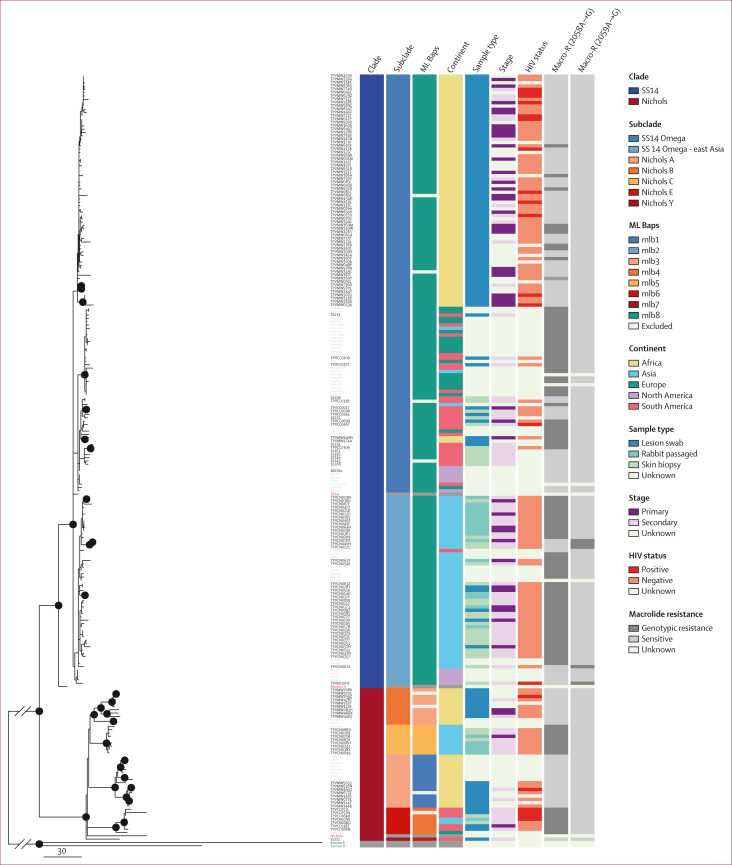
Recombination-masked TPA whole-genome phylogeny Recombination-masked TPA whole-genome phylogeny derived from 166 individuals in this study, a convenience sample of 62 genomes published in 2021,^8^ and five reference genomes (TPA [red], *Treponema pallidum* subspecies *pertenue* [blue], and *Treponema pallidum* subspecies *endemicum* [green]). Nodes with more than 80% bootstrap support are indicated with a black circle. Manual subclade assignments are included to facilitate comparison to recent published literature (subclade),^8^ alongside TPA population clusters determined using *baps* Bayesian modelling (figure 4) but with maximum likelihood phylogeny used as a prior for partitioning (ML Baps). ML=maximum likelihood.TPA=*Treponema pallidum* subspecies *pallidum.*

For participants who provided multiple specimens, WGS was conducted on samples with the highest *polA* copy number (appendix 1 pp 22–23), achieving acceptable coverage for phylogenomic analysis in 166 (95%) of 174 samples (figure 1). The 166 samples included TPA genomes from 113 (97%) of 117 participants with sufficient TPA copy numbers, 43 individuals from Malawi with DFM-negative lesions, and ten individuals from Colombia enrolled in a separate study (appendix 2 p 1).^15^ Samples collected at both visits from the re-infected participant had TPA copy numbers insufficient for WGS. In total, our phylogenomic analysis (figure 3) included 166 new genomes from this study, a convenience sample of 62 genomes published in 2021 from diverse geographical locations,^8^ and five reference genomes (233 total genomes).

**Figure 4:**
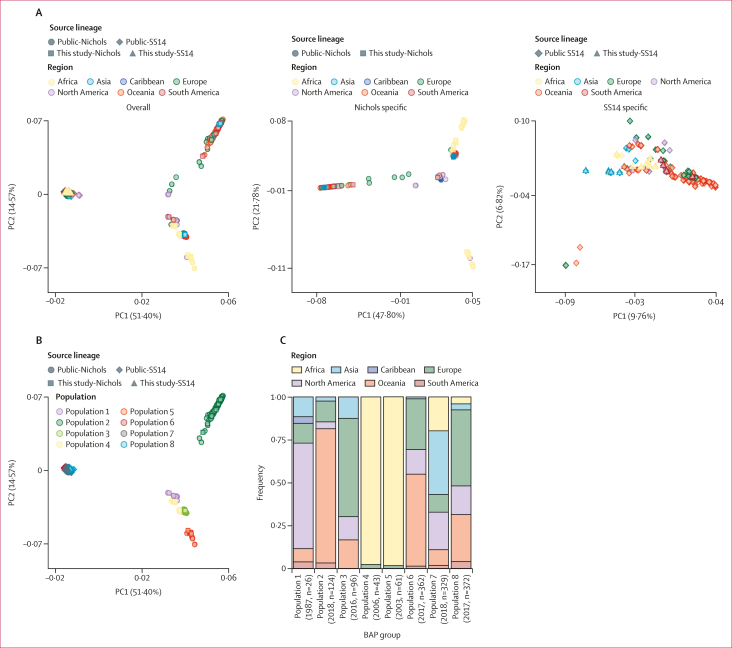
Global TPA population structure (A) PCAs of global TPA strains, overall and by lineage. Analysis of 1413 TPA genomes derived from diverse studies in global sites confirms that membership in Nichols (n=350) versus SS14 (n=1063) clade accounts for most of the genetic variation (excluding *tpr* family, *tp0470,* and *arp* genes). When PCA was restricted to members of the same lineage, more distinct population structure is evident among Nichols-like strains. (B) PCA annotated by TPA population determined using *baps* Bayesian modelling. (C) Composition of the five Nichols-lineage and three SS14-lineage populations by geography and sample collection dates. Median year of sample collection and number of samples are included. PC1=principal component 1. PC2=principal component 2. PCA=principal component analysis. TPA=*Treponema pallidum* subspecies *pallidum.*

Although most strains (133 [80%] of 166) belonged to the SS14 lineage, we observed genomically diverse strains across clinical sites (figure 3). SS14-lineage and Nichols-lineage strains were similarly distributed among groups with differing age, gender, race or ethnicity, sexual orientation and behaviours, syphilis stage, and HIV status (appendix 1 p 26); clinical manifestations did not clearly differ by lineage (figure 2). SS14-lineage strains, however, clustered by country with all Malawi SS14-lineage strains grouped within the SS14-Omega subclade (figure 3).^8^ Although only 33 (20%) of 166 genomes belonged to the Nichols clade, Nichols-lineage strains were identified across continents and diverse participants (figure 4). Compared with SS14-lineage strains, Nichols-lineage strains clustered into more distinct subclades and exhibited longer branch lengths, consistent with greater genetic divergence. Five (62%) of eight participants in the Nichols E subclade were living with HIV. This subclade consisted of strains collected from Colombia (n=5) and China (n=2), and one genome from Italy from an individual with unknown HIV status.

Macrolide susceptibility varied by geography, with 2058A→G and 2059A→G mutations in 76 (46%; 95% CI 39–54) of 166 samples with calls for both loci (figure 3). The proportion of specimens with resistance mutations ranged from 100% (95% CI 93–100) in China (n=52) and the USA (n=1), to 50% (95% CI 29–71) in Colombia (n=12) and 14% (95% CI 7–22) in Malawi (n=12), where one isolate harboured an 2058A→T allele presumed to be associated with resistance.

Principal component analysis of 1413 TPA genomes confirmed that our study strains reflect the global diversity of strains across published studies^5^, ^6^, ^7^, ^8^ (appendix 1 p 27; appendix 2 p 3). 51ꞏ4% (principal component one) of genetic variation in our recombination-masked analysis was explained by membership in the SS14 versus Nichols clades, with more distinct population structure observed in Nichols-lineage strains (figure 4A). Using Bayesian modelling, we identified eight distinct TPA populations worldwide. These populations included five Nichols-lineage (populations 1–5, n=350) and three SS14-lineage populations (populations 6**–**8, n=1063; figure 4B). Within the Nichols-lineage, one population was dominated by well characterised laboratory strains (population 1), one was dominated by Australian samples (population 2), and two (populations 4 and 5) by African samples. Samples in populations 4 and 5 originated from Madagascar (population 4 n=31 and population 5 n =52), Malawi (population 4 n=7 and population 5 n=7), and Zimbabwe (population 4 n=4 and population 5 n=1), with median collection dates before 2010 (figure 4C). TPA populations determined by Bayesian modelling were generally concordant with previously described Nichols subclades, whereas concordance with SS14 subclades was less clear (appendix 1 p 28). We identified TPA strains from all eight populations in our study, with the greatest number in the SS14-lineage population 7 and population 8 (appendix 2 p 3).

We found 103 SNVs that were fixed or absent in all SS14-lineage or Nichols-lineage strains (appendix 2 p 4), including 57 non-synonymous (missense), 30 synonymous (sense), and 16 upstream (possible promoter disrupting) SNVs. Among 344 examined biallelic SNVs, there were 217 significantly differentiated SNVs between the two lineages (Fisher’s exact p<4ꞏ451 × 10^⁻6^). These 217 biallelic SNVs included 152 non-synonymous, 48 synonymous, 16 upstream, and one stop-gained SNVs, collectively referred to as highly differentiated (appendix 2 p 5).

We observed an accumulation of non-synonymous fixed SNVs in *lptD* (*tp0515*), which encodes a homologue for the Gram-negative OMP that mediates the final step in the transport of lipopolysaccharide to the outer leaflet of the outer membrane,^9^ and in *tp0179* which encodes a hypothetical protein. The highest number of highly differentiated SNVs (including two fixed and 2626 highly differentiated non-synonymous SNVs) were observed in *tp0462*, encoding a putative lipoprotein of unknown function, followed by *tp0136,* a putative fibronectin-binding outer membrane lipoprotein. Our results also showed an accumulation of non-synonymous, highly differentiated SNVs in *tp0865* and *tp0858*, encoding two members of the FadL family of fatty acid importers, and the *tolC* homologue *tp0966*, which encodes the outer membrane factor component of an efflux pump.^8^ Overall, 12 (21%) of 57 non-synonymous fixed and 39 (26%) of 152 non-synonymous highly differentiated SNVs occurred in known or putative genes for OMPs.^9^

Geographical differences were apparent when comparing within-population allele frequency patterns by lineage and sampling location (appendix 1 p 29). Among Nichols-lineage strains, highly differentiated SNVs were common across the *tp0164* (*troB*, encoding an ATPase), *tp0462* (putative lipoprotein), and *tp0865* (*fadL*) genes with high frequencies in strains from Oceania and South America. In SS14-lineage strains, common non-synonymous SNVs included mutations in *tp0151* (*rnfD*)*, tp0515 (lptD*)*,* and *tp0705* (*mrcA*)*.* These mutations further segregated by TPA population assignment (appendix 1 p 30). SS14-lineage strains from Asia often had non-synonymous SNVs in *tp0705* (*mrcA,* encoding penicillin binding protein 1a), synonymous mutations in *tp0603* and *tp0416,* and mutations upstream of *tp0143.* Among SS14-lineage strains, the variant *mrcA* 1873A→G was common in Europe, America and Oceania, and 1517C→T was predominant in Asia. The variant 1516G→A was common in Nichols-lineage strains.

The small number of TPA genomes from Africa sequenced to-date were distinct from the global population, with unique SNV signatures. We observed two high-frequency SNVs in SS14-lineage strains from Africa, a non-synonymous mutation in *troR* (*tp0167*, allele frequency 97ꞏ5%), encoding a regulator of a transition metal uptake permease system, and a synonymous mutation in the *tp0803* gene. Several SNVs were more common among African (populations 4 and 5) than global Nichols-lineage samples, including multiple non-synonymous SNVs in the *tp0483* and *tp0136* genes encoding putative fibronectin-binding proteins. All non-synonymous SNVs in *tp0483* worldwide were exclusive to population 5. TP0136 Ser66Gly occurred at high frequency in population 5, and Asp349Glu and Ser407Leu were exclusive to population 4.

**Figure 5:**
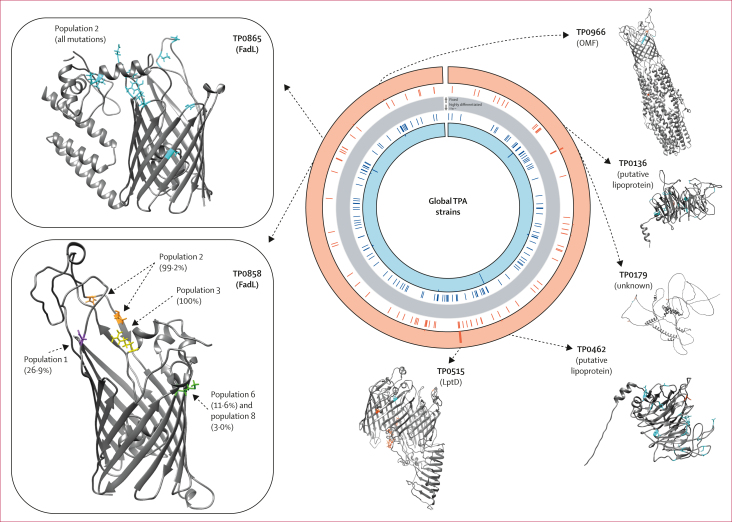
Accumulation of lineage-associated and population-associated missense mutations in select TPA proteins Evaluation of predicted protein structures for genes with multiple lineage-informative missense SNVs confirms population-specific mutations across global TPA isolates. Genes with lineage-informative fixed (orange) and highly differentiated (blue) SNVs identified during comparison of 1413 TPA genomes are highlighted in the circular plot (excluding *tpr* family, *tp0470,* and *arp* genes). Starting from the grey bar: (1) coloured lines highlight genes affected by fixed (outer; orange) or highly differentiated (inner; blue) SNVs, and (2) histograms that depict the frequency of fixed (orange background) and highly differentiated (blue background) missense mutations by gene. Nichols-lineage three-dimensional structural protein models were previously predicted by Hawley and colleagues^9^ (TP0515, TP0858, TP0865, and TP0966) or by AlphaFold2 (TP0136, TP0179, and TP0462). Boxes highlight differences by TPA population for the FadLs TP0865 and TP0858; within-population allele frequencies of more than 1% are annotated. Frequencies of lineage-informative fixed and highly informative SNVs are provided in appendix 2 (pp 4–5). Genomic coordinates correspond to the Nichols reference strain. OMF=outer membrane factor. SNV=single nucleotide variants. TPA=*Treponema pallidum* subspecies *pallidum*.

To explore the potential impact of non-synonymous SNVs on protein structures, we localised amino acid changes to three-dimensional models of proteins encoded by genes affected by the most distinct mutations (figure 5).^9^ In *lptD* (*tp0515*), nine fixed missense SNVs were present in all SS14-lineage strains and affected amino acids concentrated in its predicted C-terminal extension. Non-synonymous, highly differentiated SNVs in the FadL proteins TP0865 and TP0858 were evident in predicted extracellular loops, with TP0858 mutations segregated by a distinct TPA population. Isolates from Nichols-lineage population 2 had numerous mutations in TP0865, and two distinct mutations in TP0865, and distinct mutations in a TP0858 extracellular loop and in the β-barrel and extracellular loop of the outer membrane factor TP0966 (appendix 2 p 7). These findings confirm TPA lineage-specific and population-specific genetic variation across global populations that can alter the structures of multiple proteins.

## Discussion

Our study enrolled racially and ethnically distinct populations with early syphilis from multiple countries, of whom 24% were living with HIV, and analysed multiple specimen types that enabled analysis of genetically diverse SS14-lineage and Nichols-lineage strains. Genomic analyses of unique participant samples, identification of SNVs across TPA populations, and mapping to protein models revealed variants of periplasmic proteins and OMPs that may influence protective immunity and spirochetal virulence. To our knowledge, this is the first study to evaluate both the clinical and genomic diversity of globally circulating TPA strains in the context of efforts to develop a syphilis vaccine.

We identified TPA in samples from primary syphilis and secondary syphilis participants and found a range of qPCR results (figure 2; appendix 2 p 2). Compared with the literature,^16^, ^17^, ^18^ we observed a higher proportion of lesion swabs and skin biopsies with detectable TPA (>80%), and low PCR positivity rates from skin scrapings and whole blood. Lesion swabs from genital ulcers and condyloma lata had the highest qPCR values (ranging between 1000 and 3000 copies per μL among participants from Malawi), confirming their suitability as specimens for syphilis detection.^18^

Our TPA WGS data support findings from the literature describing syphilis genetic epidemiology,^5^, ^6^, ^7^, ^8^ and revealed several new findings. SS14-lineage strains predominated among participants with different demographic, behavioural, and clinical characteristics. Nichols-lineage strains had greater genetic divergence and clustered into more distinct subclades as previously observed.^5^^,^^6^^,^^8^ Although over half of participants infected by Nichols E subclade strains were living with HIV, these strains were distinct from the HIV-associated BP1.3 TPA sublineage (SS14 lineage) described from Australia,^7^ suggesting spread within different networks. Consistent with previous reports of widespread macrolide resistance,^14^^,^^19^, ^20^, ^21^, ^22^ most of our study sites had predominantly macrolide-resistant strains apart from Malawi. Although macrolides are no longer recommended for syphilis treatment, resistant strains remain highly prevalent suggesting that resistance might be driven by antibiotic use for other infections,^23^ or is now well established in circulating treponemal populations.

In our large-scale genomic data analysis, we identified multiple lineage-informative mutations that differentiate SS14-lineage and Nichols-lineage strains as well as TPA populations. Variation at the protein level was evident, with several TPA proteins affected by distinct mutations, including variation in OMP extracellular loops that might be driven by host immune pressure. Using TPA genomes from our study and publicly available genomes, we observed eight distinct TPA populations consistent with Leiberman and colleagues.^8^ Most of these are widely distributed, with two SS14-lineage populations (populations 7 and 8) consisting of sequences from Africa, Asia, Europe, North America, Oceania, and South America. Two Nichols-lineage populations (populations 4 and 5) were comprised almost entirely of sequences from Madagascar, Malawi, and Zimbabwe.

We observed genetic differences by lineage and geography, cataloguing hundreds of fixed and significantly differentiated SNVs that distinguish SS14-lineage versus Nichols-lineage strains. Among SS14-lineage strains from Africa, the *troR* 314A→G (Glu105Gly) mutation was highly prevalent; *troR* is a member of the *tro* operon, encoding an ATP-binding cassette importer of transition metals in TPA and *Treponema denticola*.^24^, ^25^, ^26^ Non-synonymous mutations in genes encoding periplasmic proteins (*tp0151*) and OMPs (*tp0515, tp0858, tp0865*, and *tp0966*) were highly differentiated between Nichols-lineage and SS14-lineage strains. The *rnfD* (*tp0151*) gene product belongs to the cytoplasmic membrane Rnf complex involved in oxidation of flavodoxin and generation of a transmembrane ion gradient.^27^ The *tp0515* gene encodes an OMP orthologue of the lipopolysaccharide transport system (Lpt) of Gram-negative bacteria (LptABCDEFG).^9^ In TPA, LptE is replaced by a C-terminal extension to LptD with numerous fixed mutations. We also observed multiple non-synonymous SNVs in extracellular loops of the FadLs TP0858 and TP0865 that segregated by lineage and geography. The FadL OMPs are presumably involved in importing long-chain fatty acids and are important targets for syphilis vaccine development.^9^ Our findings emphasise the importance of studying genetic variation of these and other vaccine candidate TPA proteins in diverse populations.

We also observed differences by geography in three non-synonymous variants in the *tp0705* (*mrcA*) gene coding for penicillin-binding protein 1a. Variations in the TPA *mrcA* gene have been previously identified.^7^^,^^14^^,^^28^ The biological function and clinical relevance of these variations are unclear given that penicillin remains highly effective for syphilis and confirmed resistance has never been reported.

Although our study provides an important source of TPA clinical and genomic data from under-represented areas, several limitations should be noted. First, we enrolled participants from four clinical sites, resulting in small sample sizes from each location. Thus, we could not evaluate strains in the context of sexual networks, and there was limited representation from each country. Furthermore, there were differences in recruitment strategies, resulting in dissimilar participant populations. Our data are therefore clustered, limiting our ability to compare and draw larger conclusions from participant characteristics. However, inclusion of diverse study participants improved the generalisability of our findings and was supported by genomic data confirming sampling of globally dominant TPA populations. Second, we excluded several recombinant loci shown to be under positive and purifying selection from our analysis;^29^ novel methods for resolving these loci are greatly needed.^30^ Finally, our TPA genomic analysis focused on lineage-informative variants and does not include OMP mutations subject to recombination relevant to vaccine design (eg*,* TPA repeat proteins). Additional investigations should be conducted to explore the full array of TPA protein variants and their impacts on vaccine efficacy and TPA biology.

Our multicentre study delineated clinical and genomic features of contemporary SS14-lineage and Nichols-lineage TPA infection among different populations. Although we contributed new TPA genomes from Africa and South America, more sampling from these regions is needed to define local strain diversity. We observed an array of syphilis manifestations that did not appear to differ by lineage among our participants. However, genomic analysis confirmed lineage-informative mutations that varied by TPA population. Several of these mutations localised to OMP extracellular loops and segregated by geography, confirming the importance of representative sampling across global sites. Further comprehensive analyses of TPA OMP variability within circulating strains worldwide will be essential for syphilis vaccine development.

## Data sharing

The study protocol and English case report forms are included in appendix 1. The de-identified participant REDCap database can be accessed through the UNC Dataverse at https://dataverse.unc.edu/dataverse/sena-u19. Requests to share the informed consent forms can be made by emailing the corresponding author. Raw sequencing data from this study with residual human reads removed are available through the Sequence Read Archive (BioProject PRJNA815321). Data supporting the findings of this study are available within the manuscript and appendices.

## Declaration of interests

ACS reports royalties from UptoDate; honoraria from the University of Alabama at Birmingham; and support for meetings or travel from the American STD Association as a member of the Executive Board outside the scope of the current work. JAG-L reports honoraria from the Universidad de Antioquia; support for meetings or travel from Carnott Laboratories, Cantabria Labs, Epidermique, Pharmaderm, and Janssen; receipt of writing materials from Epidermique, Cantabria labs, Isdin, Pharmaderm, Skindrugs, Loreal, Galderma, Cetaphil, Cerave, Isispharma, Carnott, Janssen, Pharmalab, Novartis, Pfizer, and Lilly outside of the scope of work. JJJ reports membership in the Worldwide Antimalarial Resistance Network. KLH reports honoraria from the Eastern Virginia Medical School and the Lawrence Livermore National Laboratory. JDR receives royalties from Biokit, Chembio, and Span Diagnostics for syphilis serodiagnostic reagents; and support for meetings or travel from Indiana University outside the scope of the current work. JBP reports research support from Gilead Sciences; non-financial support from Abbott Diagnostics; and consulting for Zymeron Corporation, all outside the scope of the current work.
